# Acceptability and Use of Portable Drinking Water and Hand Washing Stations in Health Care Facilities and Their Impact on Patient Hygiene Practices, Western Kenya

**DOI:** 10.1371/journal.pone.0126916

**Published:** 2015-05-11

**Authors:** Sarah D. Bennett, Ronald Otieno, Tracy L. Ayers, Aloyce Odhiambo, Sitnah H. Faith, Robert Quick

**Affiliations:** 1 Division of Foodborne, Waterborne, and Environmental Diseases, Centers for Disease Control and Prevention, Atlanta, GA, United States of America; 2 Epidemic Intelligence Service, Centers for Disease Control and Prevention, Atlanta, GA, United States of America; 3 Safe Water and AIDS Project, Kisumu, Nyanza Province, Kenya; The University of Iowa, UNITED STATES

## Abstract

Many health care facilities (HCF) in developing countries lack access to reliable hand washing stations and safe drinking water. To address this problem, we installed portable, low-cost hand washing stations (HWS) and drinking water stations (DWS), and trained healthcare workers (HCW) on hand hygiene, safe drinking water, and patient education techniques at 200 rural HCFs lacking a reliable water supply in western Kenya. We performed a survey at baseline and a follow-up evaluation at 15 months to assess the impact of the intervention at a random sample of 40 HCFs and 391 households nearest to these HCFs. From baseline to follow-up, there was a statistically significant increase in the percentage of dispensaries with access to HWSs with soap (42% vs. 77%, p<0.01) and access to safe drinking water (6% vs. 55%, p<0.01). Female heads of household in the HCF catchment area exhibited statistically significant increases from baseline to follow-up in the ability to state target times for hand washing (10% vs. 35%, p<0.01), perform all four hand washing steps correctly (32% vs. 43%, p = 0.01), and report treatment of stored drinking water using any method (73% vs. 92%, p<0.01); the percentage of households with detectable free residual chlorine in stored drinking water did not change (6%, vs. 8%, p = 0.14). The installation of low-cost, low-maintenance, locally-available, portable hand washing and drinking water stations in rural HCFs without access to 24-hour piped water helped assure that health workers had a place to wash their hands and provide safe drinking water. This HCF intervention may have also contributed to the improvement of hand hygiene and reported safe drinking water behaviors among households nearest to HCFs.

## Introduction

It is estimated that over half of health care facilities (HCFs) in developing countries lack access to hand washing facilities [1]. Consequently, the risk of healthcare-associated infections (HAIs) in developing countries is approximately 2–20 times greater than in higher-income countries [2]. Poor hand hygiene in healthcare facilities is a long-recognized risk factor for HAIs [3]. Additional barriers to performing adequate hand washing include healthcare worker acceptance and limited patient participation and empowerment [4–5]. Access to safe drinking water for the oral administration of medications is a related problem. Using contaminated drinking water to provide medications, including anti-tuberculosis drugs, de-worming medications, first doses of antibiotics for common infections, zinc and Vitamin A, and oral rehydration solutions can increase the risk of enteric infections in all patients and the risk of opportunistic infections in HIV-infected persons [6–7].

In recognition of these problems, the World Health Organization (WHO) / United Nations Children’s Fund (UNICEF) Joint Monitoring Programme (JMP) have proposed for the post-2015 Sustainable Development Goals that by 2030 all HCFs should have an improved water source and hand washing facilities that have both water and soap available for hand washing near food preparation, sanitation, and patient care areas [8]. To achieve these targets, considerable investments in infrastructural improvements at HCFs will be needed. Therefore, in the short to medium term, affordable and effective solutions are needed to protect the health of patients and staff through improved access to hand washing facilities and safe drinking water in HCFs. One potential solution that has proven feasible includes installation of inexpensive, portable, hand washing and safe drinking water stations; distribution of starter supplies of soap and water treatment products; and healthcare worker training on safe water and hand hygiene [9–10].

In 2010, the Kenyan Ministry of Health (KMOH), Christian Health Association (CHAK), UNICEF, and the Safe Water and AIDS Project (SWAP—a Kenyan non-governmental organization) implemented a similar clinic-based intervention in western Kenya. The objective of this study was to evaluate the use and acceptability of installed portable hand washing and drinking water stations, and assess their impact on healthcare worker knowledge, and adoption of safe drinking water and hand hygiene practices by households in the surrounding community.

## Materials and Methods

### Program design

**Fig 1:**
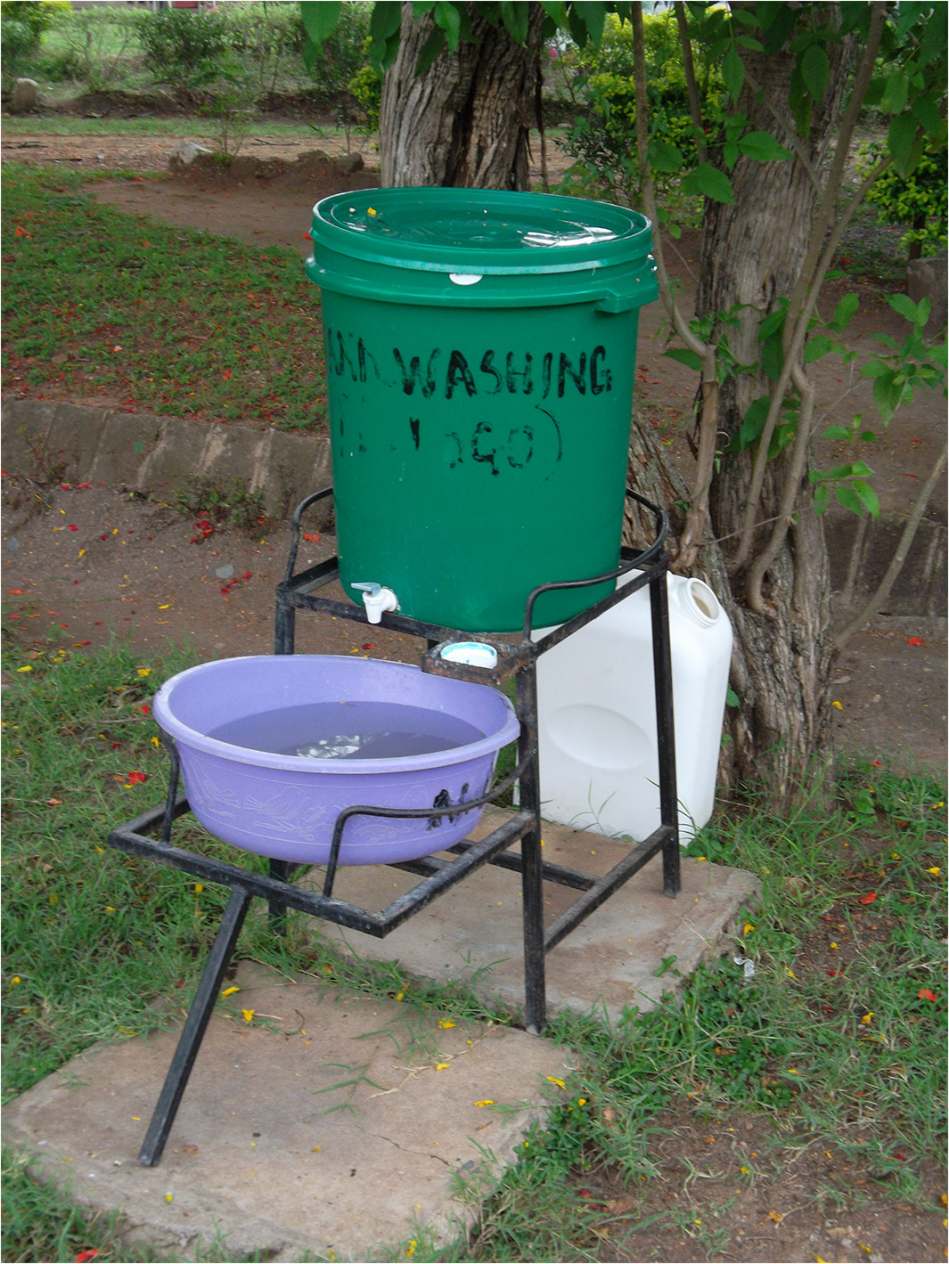
Hand Washing Station.

**Fig 2:**
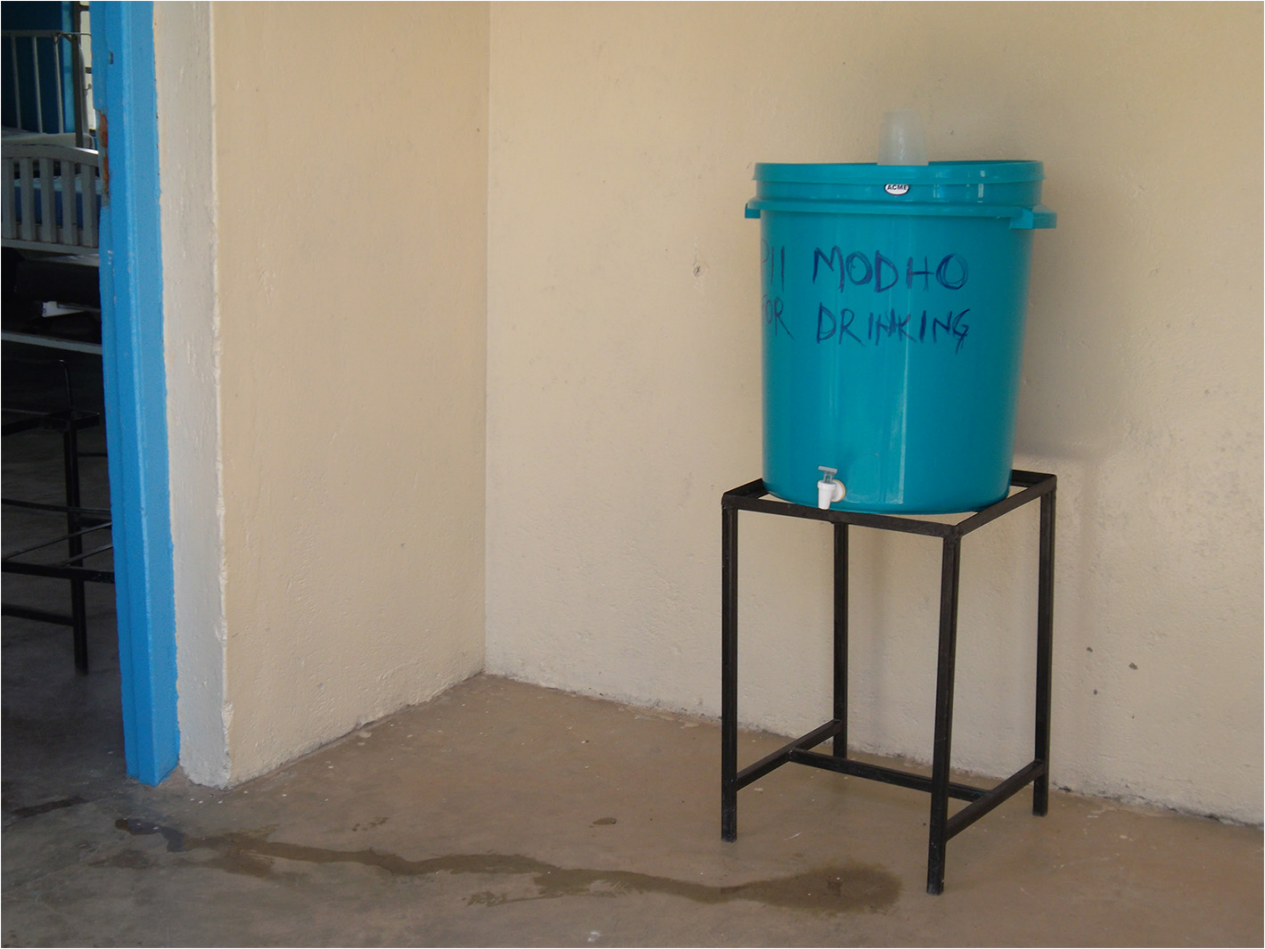
Drinking Water Station.

**Fig 3:**
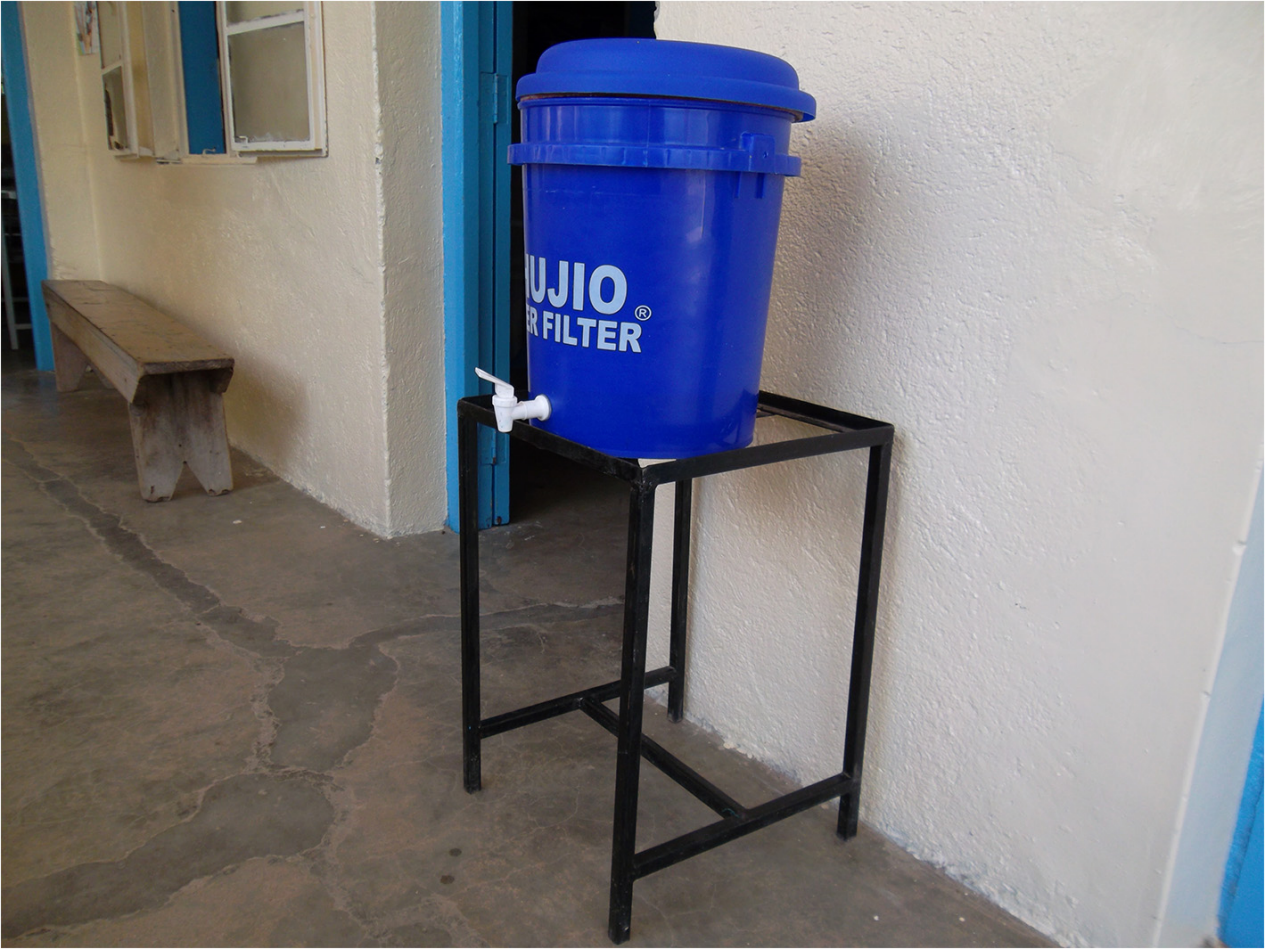
Ceramic (Chujio) Water Filter.

UNICEF selected 5 districts in western Kenya for the program— usia, Bondo, Nyando, Rachuonyo, and Homa Bay—that had experienced frequent flooding, high rates of diarrheal illness, and recent cholera outbreaks. All HCFs received two types of water stations. For hand washing, health facilities received a 60-liter bucket with lid and spigot; a basin; a metal frame with a soap receptacle (Fig 1); and a starter supply of hand soap. Drinking water stations consisted of either a 60 liter bucket with lid and spigot on a metal stand (Fig 2), accompanied by a “starter” supply of WaterGuard sodium hypochlorite solution and/or Aquatabs (sodium dichloroisocyanurate tablets) and/or PuR (flocculant-disinfectant powder), or a porous, silver-impregnated ceramic pot filter inside a 20 liter water storage bucket with lid and spigot on a metal stand (hereafter referred to as chujio filters; Fig 3). All 40 health facilities received a supply of WaterGuard solution; 37 (92.5%) received chujio filters, including all 4 hospitals, all 5 health centers, and 28 (90.3%) of 31 dispensaries. Before delivery and installation of drinking and hand washing stations, two healthcare workers from each district attended a CHAK-sponsored training led by SWAP. Training focused on drinking water treatment and storage, hand hygiene, and techniques to promote behavior change among colleagues and patients.

### Evaluation design

In February 2011, we conducted a baseline evaluation at 40 health facilities in the five districts. Following baseline data collection, SWAP, CHAK, and the KMOH implemented the program from March through November 2011. A follow-up evaluation was performed in May 2012. The baseline and follow-up evaluations each had three components: (1) HCF survey; (2) healthcare worker self-assessment; and, (3) household survey.

### Enrollment

HCFs were randomly selected proportional to the number of eligible KMOH and CHAK-sponsored dispensaries (the lowest level and first line of contact with the health care system, providing mainly preventive and minor curative ambulatory services), health centers (which provide preventive and curative health services, usually with some capacity for inpatient care), and hospitals (which provide a wide range of inpatient and outpatient services) in each of the five districts. HCF eligibility to participate in the evaluation was determined via telephone interviews; facilities were excluded if at baseline they had access to piped water 24 hours per day or had an improved hand washing station with a tap.

All healthcare workers present in rural dispensaries and health centers and all outpatient healthcare workers in hospitals on the day of the HCF evaluation were asked to participate in the health worker self-assessment.

All households with children under 1 year of age and within the census enumeration area nearest to the HCF were enrolled in the household survey. Community health workers and HCF volunteers helped enumerators locate eligible households.

### Data collection

#### Health facilities

At baseline, we conducted surprise visits and interviewed the HCF director to determine the primary water source, use of water treatment products, and presence of water treatment products and soap. We observed the types of hand washing and drinking water stations present in the facility. We defined a handwashing station as “adequate” if it consisted of a washbasin with soap present. “Improved” handwashing stations were defined as a water container with a tap and a basin with soap present. All available drinking water was tested for free residual chlorine (FRC) using the N,N diethyl-p-phenylene diamine (DPD) method (Lamotte Co., Chestertown, MD). Similar procedures and instruments were used for follow-up interviews.

#### Health workers

On the day of the baseline HCF visit, health workers were asked to complete a questionnaire that focused on knowledge about water treatment, water storage, and hand hygiene, and related patient education practices. At follow-up, a similar questionnaire was used. Because of high staff turnover, it was not possible to collect self-assessments from the same healthcare workers at baseline and follow-up.

#### Households

On the day of the baseline HCF visit, households were visited and interviews focused on household water sources, and hygiene and water handling knowledge and practices were conducted with the female caretaker of children. Observations of hand washing stations and water storage containers were made, and caretakers were asked to demonstrate their hand washing technique. Available stored drinking water was tested for FRC. The same women were surveyed at follow-up with a similar instrument; only women interviewed at both baseline and follow-up were included in the final analysis.

### Ethical review

The evaluation protocol was approved by the Kenya Medical Research Institute Ethical Review Committee (protocol 1953). The Institutional Review Board of the Centers for Disease Control and Prevention determined that, because the evaluation examined a proven public health practice, it was not research and did not require IRB review. Consent for HCF participation was obtained from the Provincial and District Health Offices. Written informed consent was obtained from all evaluation participants. Personal identifiers were permanently removed from databases following the completion of follow-up data collection.

### Data analysis

Data were entered into a Microsoft Access 2007 (Redmond, WA, USA) database and analyzed in SAS version 9.3 (Cary, NC, USA). HCF and household baseline and follow-up data were analyzed using McNemar’s test for paired proportions. In a few instances where the McNemar’s test was not feasible, an exact test of a binomial proportion was used. Because small numbers of health centers and hospitals were enrolled in the HCF evaluation, p-values are only reported for dispensary data. Health worker survey data were analyzed descriptively. For the purposes of the HCF and household analysis, improved water sources included piped water, boreholes, rain water catchment, public taps, and protected wells; all other sources of water were classified as unimproved. An improved drinking water station was defined as either a bucket with tight fitting lid and spigot or a chujio water filter. Safe drinking water was defined as either a bucket with lid, spigot and detectable FRC or a chujio water filter. A hand washing station was considered adequate, but not improved, if the water supply was not dispensed through a tap, but a soap and basin were present.

## Results

### Health facilities

A total of 40 health facilities were enrolled at baseline: 4 hospitals, 5 health centers, and 31 dispensaries. These facilities collectively served an estimated 2,200 outpatients per day (range 10–300 per facility). During the baseline assessment, 32 (80%) of 40 HCFs reported using improved water sources, including rain water catchment (50%), boreholes (34%), piped water (13%), and public taps (3%). Unimproved water sources reported by the remaining 8 (20%) of HCFs, included surface water (75%) and unprotected wells (25%). Water sources did not change at follow-up. Additionally, 17 (43%) HCF directors reported that the main water source was not on HCF grounds and 6 (15%) reported that the time required to collect water was greater than 30 minutes. Off-site water sources were used by 48% of dispensaries, 40% of health centers, and no hospitals. Among 40 HCF directors, 17 (43%) reported having a budget for the purchase of soap; 6 also had a budget for water treatment products.

Adequate hand washing stations were observed in 21 (53%) of 40 facilities at baseline and improved stations were observed in 31 (78%) at follow-up, with a statistically significant increase among dispensaries (42% vs. 77%, p = 0.01). Of 40 HCFs, 31 (78%) at baseline and 37 (93%) at follow-up reported treating stored drinking water (Table 1); among the 31 dispensaries, the increase was statistically significant (74% vs. 97%, p = 0.02). WaterGuard was the most common treatment method reported, both at baseline (68%) and follow-up (88%). Filtration of drinking water was reported by no facilities at baseline and by 8 (20%) at follow-up; a number of health providers might not have understood that chujio filters were, in fact, filtration devices. In dispensaries, there was a significant increase from baseline to follow-up in reported use of WaterGuard (61% vs. 90%, p = 0.01) and filtration (0% vs. 19%, p<0.01). The number of HCFs with at least one improved drinking water station increased from 7 (18%) at baseline to 39 (98%) at follow-up, with a statistically significant increase among dispensaries (19% to 97%, p<0.01). In HCFs with water available for testing, FRC was detected in at least one drinking water station at 9 (26%) of 35 facilities at baseline and 3 (8%) of 36 at follow-up. However, because of observed use of chujio filters in 22 HCFs, the number of HCFs with safe drinking water increased from 2 (5%) of 40 HCFs at baseline to 23 (58%) at follow-up; the increase in access to safe water among 31 dispensaries from 2 (6%) at baseline to 17 (55%) at follow-up was statistically significant (p<0.01). Although WaterGuard was the most reported water treatment method, observations at follow-up indicated that the most commonly adopted treatment method was filtration, and that filtration was largely responsible for the increase in the availability of safe water.

**Table 1:** Drinking water and hand washing observations at 40 health facilities at baseline and follow-up, western Kenya, 2011 and 2012.

	Dispensaries (N* = 31)				Health centers (N* = 5)				Hospitals (N* = 4)				Total (N* = 40)			
	Baseline		Follow-up		Baseline		Follow-up		Baseline		Follow-up		Baseline		Follow-up	
	No.	(%)	No.	(%)	No.	(%)	No.	(%)	No.	(%)	No.	(%)	No.	(%)	No.	(%)
Treats stored drinking water with any method	23	(74)	30	(97)	5	(100)	4	(80)	3	(75)	3	(75)	31	(78)	37	(93)
WaterGuard	19	(61)	28	(90)	5	(100)	4	(80)	3	(75)	3	(75)	27	(68)	35	(88)
PuR	3	(10)	4	(13)	0	(0)	1	(20)	1	(25)	0	(0)	4	(10)	5	(13)
Filtration†	0	(0)	6	(19)	0	(0)	2	(40)	0	(0)	0	(0)	0	(0)	8	(20)
Boiling	3	(10)	0	(0)	0	(0)	0	(0)	0	(0)	0	(0)	3	(8)	0	(0)
**Observations**																
≥1 improved DWS†	6	(19)	30	(97)	0	(0)	5	(100)	1	(25)	4	(100)	7	(18)	39	(98)
Bucket, lid, spigot†	6	(19)	27	(87)	0	(0)	4	(80)	1	(25)	4	(100)	7	(18)	35	(88)
Chujio water filter	0	(0)	16	(52)	0	(0)	4	(80)	0	(0)	2	(50)	0	(0)	22	(55)
≥1 DWS with detectable FRC‡	7	(26)	3	(11)	1	(25)	0	(0)	1	(25)	0	(0)	9	(26)	3	(8)
≥1 safe DWS† §	2	(6)	17	(55)	0	(0)	4	(80)	0	(0)	2	(50)	2	(5)	23	(58)
≥1 HWS with soap and basin†	13	(42)	24	(77)	5	(100)	4	(80)	3	(75)	3	(75)	21	(53)	31	(78)

Abbreviations: No. = Number, DWS = Drinking water station, FRC = Free residual chlorine, HWS = Hand washing station.

*For some items, N may vary by small numbers.

†P<0.05 by McNemar's test of dispensaries only.

‡Among facilities with water available for testing.

§Safe drinking water station defined as a bucket with lid and spigot and detectable free residual chlorine or a chujio water filter.

### Health workers

Healthcare workers at 37 (93%) of 40 facilities completed baseline (n = 67) and follow-up (n = 55) self-assessments (Table 2). At follow-up, 13 (24%) of 55 respondents reported that they had been employed less than 12 months at their current facility. At follow-up, a higher percentage of healthcare workers than at baseline reported receiving formal training or informal training by a colleague on hand washing, water treatment, and water storage (80% vs. 25%) and teaching their clients about these topics (93% vs. 66%). At baseline and follow-up, over 67% of healthcare workers exhibited knowledge about when hand washing should be performed, the correct dosing and contact time of WaterGuard and Aquatabs, and the best types of safe water storage containers.

**Table 2:** Healthcare worker characteristics, training, and patient education practices, and knowledge regarding water treatment, water storage and hygiene principles at baseline and follow-up, western Kenya, 2011 and 2012.

	Baseline		Follow-up	
	N* = 67		N* = 55	
	No.	(%)	No.	(%)
Median age (range), years	30	(24–57)	34	(24–60)
Male gender	23	(35)	20	(36)
Employed at current HCF>12 months	64	(100)	42	(76)
Trained on water treatment, storage, hand hygiene	17	(25)	44	(80)
Formal training	5	(8)	28	(51)
Informal training by a colleague	16	(24)	41	(75)
Reported teaching patients about water treatment, storage, hand hygiene	44	(66)	51	(93)
**Knowledge of water treatment and water storage**				
All water sources should be treated	48	(72)	40	(73)
Correct dose of WaterGuard or Aquatabs	62	(93)	48	(87)
Correct contact time of WaterGuard or Aquatabs	52	(80)	46	(85)
Identified characteristics of safe water storage containers	46	(69)	43	(78)
**Knowledge of hand hygiene**				
Identified when hand washing should be performed				
Before eating	64	(96)	51	(93)
Before food preparation	52	(78)	52	(95)
After visiting the toilet	65	(97)	52	(95)
After cleaning a child who has defecated	53	(79)	49	(89)
When hands are dirty	52	(78)	48	(87)
After coughing, sneezing, or blowing nose	46	(69)	47	(85)
All identified	48	(72)	41	(75)
Identified steps in correct hand washing				
Use both soap and water	66	(99)	52	(95)
Rub hands together	58	(87)	47	(85)
Rub between fingers	60	(90)	50	(91)
Clean under fingernails	52	(78)	48	(87)
Rinse hands	55	(82)	48	(87)
Dry with clean towel	45	(67)	37	(67)
If no clean towel available, air dry hands	46	(69)	42	(76)
All steps identified	37	(55)	27	(49)

Abbreviations: HCF = health care facility.

*For some items, N may vary by small numbers.

### Households

We enrolled 566 households at baseline, and 391 (66%) at follow-up. A higher proportion of respondents in households lost to follow-up were single (15% vs. 6%, p<0.01) and reported using improved sources of water (47% vs. 35%, p = 0.01) than at baseline; otherwise the two groups were similar. Households lost to follow-up were omitted from data analysis.

At baseline, the median age of female respondents was 24 years (interquartile range (IQR) 20–28); 92% were married and 48% reported having completed less than a complete primary school education. Households were composed of a median of 5 household members (IQR 4–7 members), with a median of 2 children (IQR 1–2) under 5 years of age. Only 3% of households had electricity. The most commonly reported household assets were radios (75%), telephones (62%), and bicycles (51%).

Reported household use of improved water sources significantly increased from baseline to follow-up (36% vs 44%, p<0.01) (Table 3). Overall, the most common household water sources reported at baseline were surface waters (50%), protected wells (14%), and protected springs (10%); and at follow-up, were surface waters (46%), protected wells (25%), and public taps (9%). A similar percentage of households at baseline (37%) and follow-up (36%) were observed to have at least one improved drinking water storage container. Significantly more household respondents reported treatment of stored drinking water at follow-up than at baseline (92% vs. 73%, p<0.01). Reported use of WaterGuard or Aquatabs was the most common treatment method reported at baseline and follow-up; reported use of WaterGuard or Aquatabs, boiling, and filtration all significantly increased during the study period. Among households with stored water available for testing, FRC was detected in 6% of samples at baseline and 8% at follow-up.

**Table 3:** Household knowledge, attitudes and practices regarding water treatment and water storage at baseline and follow-up, western Kenya, 2011 and 2012.

	Baseline		Follow-up	
	N* = 391		N* = 391	
	No.	%	No.	%
**Water storage knowledge**				
Learned about water storage at HCF†	138	(35)	227	(59)
Identified best type of water storage container†	242	(62)	279	(71)
**Waterguard or Aquatabs knowledge**				
Learned about WaterGuard or Aquatabs at HCF†	174	(45)	262	(67)
Knew correct dose of WaterGuard or Aquatabs†	290	(75)	354	(91)
Knew correct contact time of WaterGuard or Aquatabs	254	(66)	277	(71)
**Household drinking water practices and observations**				
Uses improved drinking water source† §	140	(36)	171	(44)
Observed ≥1 improved water storage container	146	(37)	137	(36)
Treats stored drinking water with any method†	285	(73)	359	(92)
Waterguard or Aquatabs†	228	(58)	289	(74)
Boiling†	75	(19)	103	(26)
Filtration†	39	(10)	115	(29)
Other	17	(5)	17	(5)
Ever used WaterGuard or Aquatabs†	331	(85)	356	(91)
Detected FRC in stored water	20	(6)	27	(8)

Abbreviations: No. = Number, HCF = Health care facility, FRC = Free residual chlorine.

*For some items, N may vary by small numbers.

†P<0.05 by McNemar's test.

‡P<0.05 by exact test of binomial proportion.

§Improved drinking water sources include piped water, boreholes, public taps and protected wells or springs.

There was a significant increase in the number of household respondents who reported learning about hand hygiene, water treatment, and water storage at a HCF during the study period (Table 3 and Table 4). Household respondents were also significantly more likely to correctly identify target times for hand washing, know the correct dose of WaterGuard or Aquatabs, and identify the best types of water storage containers at follow-up compared with baseline. A higher percentage of household respondents at follow-up than baseline performed all four hand washing steps correctly (43% vs 32%, p = 0.01).

**Table 4:** Household knowledge, attitudes and practices regarding hand hygiene at baseline and follow-up, Nyanza and Western Provinces, Kenya, 2011 and 2012.

	Baseline		Follow-up	
	N* = 391		N* = 391	
	No.	%	No.	%
**Hand washing knowledge**				
Learned about hand washing at HCF†	206	(53)	323	(83)
Identified when hand washing should be performed				
Before eating†	250	(64)	309	(79)
Before food preparation†	90	(23)	124	(32)
After visiting toilet†	314	(80)	368	(94)
After cleaning a child who has defecated†	106	(27)	188	(48)
All correct†	16	(4)	49	(13)
Knew correct hand washing time†	40	(10)	136	(35)
**Household hygiene and hand washing practices and observations**				
Steps performed on hand washing demonstration				
Uses soap and clean water†	312	(80)	329	(92)
Lathers all surfaces†	267	(69)	304	(85)
Rinses hands†	319	(82)	352	(98)
Dries hands with clean towel or air dries†	157	(40)	177	(49)
All steps demonstrated†	125	(32)	154	(43)

Abbreviations: No. = Number, HCF = Health care facility.

*For some items, N may vary by small numbers.

†P<0.05 by McNemar's test.

‡P<0.05 by exact test of binomial proportion.

## Discussion

The findings of this evaluation demonstrated that low-cost, low-maintenance, locally-available, portable hand washing and drinking water stations were acceptable short- to medium-term interventions to assure that health workers had a place to wash their hands and provide safe drinking water for medication administration. The presence of these stations in HCFs and the emphasis on patient teaching also appeared to contribute to the improvement of hand hygiene and safe drinking water behaviors among households nearest to HCFs.

The use of the water stations by health personnel was anticipated, and has been observed in other evaluations [9–10]. Since 1847, poor hand hygiene has been known to be a contributing risk factor for the occurrence of HAI’s [3]. Healthcare workers surveyed in this study demonstrated adequate knowledge of hand hygiene behaviors as well as knowledge about drinking water treatment and storage. This level of knowledge was high, even at baseline when few facilities had adequate hand washing stations with soap and water, water storage containers, and water treatment products. Providing HCFs with these supplies, coupled with high levels of healthcare worker knowledge, likely contributed to the successful adoption and continued use of these stations in many HCFs. The apparent lack of use of improved water stations in some health facilities likely resulted from several barriers to adoption, including staff turnover, lack of funding for purchasing soap and water treatment products, and time needed to collect water from distant sources.

The modest improvements in hand washing and water handling behaviors in households located near project HCFs have been observed in other studies [9,11–13] and could be explained by several factors. First, at follow-up, an increased percentage of health personnel reported having been trained on these topics and teaching them to their clients. Health personnel reports were corroborated by an increase in the percentage of HCF clients who reported hearing about hand washing technique, safe water storage, and water treatment at the nearby HCF. Second, health personnel have been shown to be a credible source of health information, raising the possibility that their teaching was translated into action by some of their patients [9, 10, 14]. Third, HCF clients were taught about a range of locally-available water treatment technologies which may have increased the likelihood of meeting consumer demand for particular products [15]. Although few households were found to have drinking water containers with detectable residual chlorine, suggesting that barriers inhibited adoption of drinking water chlorination in the home, there was a significant increase in the reported use of filters. The discrepancy between reported and observed chlorine use, and the implied barriers to current use, has been observed in other studies [16, 17].

Similarly, distribution of ceramic water filters may have contributed to the low adoption of chlorine-based water treatment products at HCFs. In fact, over half of facilities at follow-up were observed to be using filters for the treatment of drinking water, while few had detectable residual chlorine in stored drinking water containers, suggesting a preference for filtration over the use of chlorine-based water treatment products. Ceramic water filters, with proper care, can last 2 or more years with no recurring costs for water treatment products [18]. The low operating costs of filters and their ease of use are two reasons that households reported preferences for the use of ceramic water filters over consumable water treatment products, including WaterGuard and PuR, in a recent study [15]. To our knowledge this is the first study to evaluate the use of ceramic water filters in HCFs where budgets for the purchase of consumable water treatment products is limited and busy health personnel may not have time to adhere to the multiple steps recommended for adequate treatment. Ceramic water filters may not be ideal for all HCFs or patient care areas, especially where large volumes of treated water may be needed; in fact, chujio filters were observed to be in use in only 16 (57.1%) of 28 dispensaries and 4 (80%) of 5 health centers.

This evaluation had several limitations. First, the long-term impact of this clinic-based intervention cannot be determined after a single 12- to 15-month observational period. However, a previous study suggested that HCFs continue to use drinking water and hand washing stations up to 4 years after installation [10]. Providing HCFs with alternative methods for water treatment and water storage, both for drinking and hand washing, will likely increase long-term adoption of safe drinking water and hand hygiene behaviors in health care facilities. Second, because communities nearest to the selected HCFs were evaluated, it is not known whether this intervention would have similar impacts on communities further away. Third, because of financial and time constraints, we were unable to test water for microbial contamination as a definitive test of drinking water quality. Finally, financial and time constraints did not permit an evaluation of health outcomes attributable to the intervention, either at the HCF or in the community; future studies should measure the impact of these interventions on HAI’s and on diarrheal diseases in the community.

## Conclusions

In conclusion, in a region where over half of health facilities lack water supplies onsite and none had reliable 24-hour access, this simple, inexpensive intervention served an immediate need for hand washing and drinking water in HCFs in Kenya. The critical importance of access to hygiene infrastructure as the first line of defense against disease has been underscored by the risks faced by health personnel in Ebola-affected West African countries working in HCFs without hand washing stations [19]. In recognition of the neglected crisis of access to water, sanitation, and hygiene in HCFs in the developing world, WHO and UNICEF have recommended universal coverage of water and sanitary infrastructure in HCFs by 2030 as a Sustainable Development Goal that will follow the conclusion of the Millennium Development Goal initiative in 2015 [8]. Clinic-based installation of portable hand washing and drinking stations can provide a short- to medium-term solution to protect health while the longer process of construction of water and sanitary infrastructure is underway. In HCFs that lack an improved water supply 24 hours a day because of water scarcity, these stations could become a more permanent fixture.
